# Dystroglycan Mediates Clustering of Essential GABAergic Components in Cerebellar Purkinje Cells

**DOI:** 10.3389/fnmol.2020.00164

**Published:** 2020-08-28

**Authors:** Federica Briatore, Giulia Pregno, Silvia Di Angelantonio, Elena Frola, Maria Egle De Stefano, Cyrille Vaillend, Marco Sassoè-Pognetto, Annarita Patrizi

**Affiliations:** ^1^Department of Neuroscience “Rita Levi Montalcini”, University of Turin, Turin, Italy; ^2^Department of Physiology and Pharmacology, Sapienza University of Rome, Rome, Italy; ^3^Center for Life Nanoscience, Istituto Italiano di Tecnologia, Rome, Italy; ^4^Department of Biology and Biotechnology “Charles Darwin”, Istituto Pasteur Italia-Fondazione Cenci Bolognetti, Sapienza University of Rome, Rome, Italy; ^5^CNRS, Institut des Neurosciences Paris-Saclay, Université Paris-Saclay, Gif-sur-Yvette, France; ^6^Schaller Research Group Leader at the German Cancer Research Center, Heidelberg, Germany

**Keywords:** neuroligin 2, GABA_A_ receptors, cell adhesion molecules, dystrophin, synapse organizer

## Abstract

Muscle dystrophin–glycoprotein complex (DGC) links the intracellular cytoskeleton to the extracellular matrix. In neurons, dystroglycan and dystrophin, two major components of the DGC, localize in a subset of GABAergic synapses, where their function is unclear. Here we used mouse models to analyze the specific role of the DGC in the organization and function of inhibitory synapses. Loss of full-length dystrophin in *mdx* mice resulted in a selective depletion of the transmembrane β-dystroglycan isoform from inhibitory post-synaptic sites in cerebellar Purkinje cells. Remarkably, there were no differences in the synaptic distribution of the extracellular α-dystroglycan subunit, of GABA_A_ receptors and neuroligin 2. In contrast, conditional deletion of the dystroglycan gene from Purkinje cells caused a disruption of the DGC and severely impaired post-synaptic clustering of neuroligin 2, GABA_A_ receptors and scaffolding proteins. Accordingly, whole-cell patch-clamp analysis revealed a significant reduction in the frequency and amplitude of spontaneous IPSCs recorded from Purkinje cells. In the long-term, deletion of dystroglycan resulted in a significant decrease of GABAergic innervation of Purkinje cells and caused an impairment of motor learning functions. These results show that dystroglycan is an essential synaptic organizer at GABAergic synapses in Purkinje cells.

## Introduction

Synapse formation is a key step in the development of neuronal networks. Research over the past few decades has led to the identification of several factors that play a role in the assembly, maturation and remodeling of synaptic connections, and provide a basis for the molecular and functional specificity of synapses (Shen and Scheiffele, 2010; Yogev and Shen, 2014). It is believed that cell type-specific formation of a nascent synapse and its subsequent maturation, involving the assembly of pre- and post-synaptic signaling machinery, are mainly mediated by synaptic cell-adhesion molecules (Yamagata et al., 2003). Interestingly, a substantial number of different synaptic cell-adhesion systems have been shown to control the formation of glutamatergic synapses, through specific PDZ-binding domains (Siddiqui and Craig, 2011). In contrast, GABAergic post-synaptic compartments comprise very few PDZ-domain-containing proteins and instead require gephyrin, a scaffolding protein, to accumulate GABA_A_ receptors (GABA_A_Rs) (Tyagarajan and Fritschy, 2014). GABAergic synapses also contain selective cell-adhesion proteins, such as neuroligin 2 (NL2) (Varoqueaux et al., 2004), and in some cases the dystrophin–glycoprotein complex (DGC) has been shown to play a role in inhibitory synaptic function (Anderson et al., 2003; Kueh et al., 2008; Pribiag et al., 2014).

The DGC is a large, membrane-spanning protein complex that links the extracellular matrix to the actin-associated cytoskeleton in both skeletal muscle and non-muscle tissues (Barresi and Campbell, 2006). The DGC can be resolved into three classes of proteins: (1) α and β-dystroglycan (DG), (2) the cytoplasmatic subcomplex composed by dystrophin and dystrobrevin, and (3) the sarcoglycan complex (Blake et al., 2002). Studies on brain have revealed that the DGC is expressed both in glia and in neurons, and is localized post-synaptically in a subset of inhibitory synapses, where its stoichiometric composition and function remain poorly characterized (Blake et al., 1999; Moukhles and Carbonetto, 2001).

The importance of the DGC for brain function is testified by clinical observations revealing that dystrophies, a group of muscular diseases driven by mutation of dystrophin, and dystroglycanopathies, a heterogenous group of muscular dystrophies caused by hypoglycosylation of α-DG with O-linked carbohydrates (Martin, 2005), are frequently accompanied by cognitive impairments and epilepsy with or without structural brain abnormalities (Godfrey et al., 2011; Devisme et al., 2012).

Dystroglycan is present in a subset of GABA synapses in forebrain neurons and cerebellar Purkinje cells (PCs), where it co-localizes with other members of the GABAergic post-synaptic specialization (Levi et al., 2002; Grady et al., 2006; Briatore et al., 2010). It comprises two subunits, the extracellular α-DG and the transmembrane β-DG, derived from post-translational cleavage of a precursor polypeptide (Ibraghimov-Beskrovnaya et al., 1992). The α subunit is heavily glycosylated and binds with high affinity to laminin and other laminin G (LG)-like domain-containing molecules, such as agrin, perlecan and pikachurin, via O-linked sugar chains associated with its central mucin domain (Barresi and Campbell, 2006; Muntoni et al., 2007; Goddeeris et al., 2013). The β subunit has a single transmembrane domain that binds dystrophin at its cytoplasmic tail and extracellularly interacts with α-DG (Ervasti and Campbell, 1993). Biochemical studies have revealed that at synapses the α/β-DG complex can interact both with presynaptic adhesion proteins, such as NRX and NRX-like family components (Sugita et al., 2001; Siddiqui and Craig, 2011), and with post-synaptic intracellular scaffolds, such as S-SCAM, a member of the membrane-associated guanylate kinase (MAGUK) family of PDZ-domain-containing proteins. In turn, the PDZ domain of S-SCAM interacts with the C-terminal tail of NL2, linking the NRX-NL adhesion system with the DGC (Sumita et al., 2007). Thus, α/β-DG binds to essential extracellular and intracellular synaptic components, supporting the idea that it is a suitable candidate as a mediator of synaptic specificity (Sassoe-Pognetto and Patrizi, 2017). However, the role of DG in *trans*-synaptic signaling is poorly characterized (Sugita et al., 2001; Früh et al., 2016).

In the present study, we explored the role of DG in GABAergic synapse organization in PCs. We show that DG is required for post-synaptic localization of NL2, GABA_A_Rs and S-SCAM. Deletion of DG causes a severe reduction of GABAergic innervation of PCs, and affects motor learning, indicating that GABAergic synapses are critically dependent on DG *in vivo*. Furthermore, comparison of conditional DG knockout (KO) mice with *mdx* mice lacking full-length dystrophin indicates that the extracellularly-located α-DG acts as a major organizer of GABAergic synapses. These results suggest that *trans*-synaptic interactions mediated by α-DG regulate the organization and maintenance of GABAergic synapses in cerebellar PCs.

## Materials and Methods

### Mice

Adult *mdx* mice (C57BL/10ScSn-Dmd*^*mdx*^*/J) lacking dystrophin and C57BL/10J controls were used in the study (Vaillend et al., 2004). Mice carrying a floxed version of the *Dag1* gene (Cohn et al., 2002) were purchased from the Jackson laboratory (129-Dag1^TM 2*Kcam*^/J, # 006835). The homozygous mice were crossed with mice hemizygous for L7Cre transgene (Barski et al., 2000). Littermates of the following genotypes were used for the experiments: *Dag1*lx/*Dag1*lx/L7Cre (PC-ΔDG) and *Dag1*lx/*Dag1*lx (c-WT). Briefly, mice were genotyped by PCR analysis of genomic DNA from biopsies using the following primer pairs: DG1 (5′-GGAGAGGATCAATCATGG-3′) plus DG2 (5′-CAACTGCTGCATCTCTAC-3′) to test for the *Dag1* allele (516 bp band for wt, 615 bp band for mutant); Cre1 (5′-GACCAGGTTCGTTCACTCATGG-3′) plus Cre2 (5′-AGGCTAAGTGCCTTCTCTACAC-3′) to test for the Cre recombinase transgene (250 bp band for L7Cre).

The experimental procedures were designed in accordance with national (Legislative Decree 116/92 and law n. 413/1993) and international (Directive 86/609/EEC and the recommendation 2007/526/EC from the Europen Community) laws and policies, and approved by the Italian Ministry of Health (Department of Public Veterinary Health) and by the ethical committee of Turin University. All efforts were made to minimize the animal suffering and the number of animals used.

### Immunofluorescence

For detection of post-synaptic molecules, we used the protocol described in Patrizi et al. (2008). Briefly, mice were anesthetized and decapitated, the brains were excised and the cerebellum was cut manually in sagittal slabs that were fixed by immersion in ice-cold formaldehyde (4% in 0.1M phosphate buffer, PB, pH 7.4) for 30 min. For detection of presynaptic molecules, mice were perfused with 4% formaldehyde in PB, and their brains were post-fixed overnight. Tissue slabs were cryoprotected in sucrose, sectioned with a cryostat, and the sections were collected on gelatin-coated slides. Following a blocking step in normal goat or donkey serum (3% in PBS with 0.5% Triton X-100), the sections were incubated overnight with a combination of the following antibodies: anti-GABA_A_Rα1 (rabbit, 1:5000) and anti-GABA_A_Rγ2 (guinea pig, 1:2000) kindly provided by Dr. J.-M. Fritschy (University of Zurich, Switzerland); anti-neuroligin 2 (NL 2) (rabbit, 1:2000) kindly provided by Dr. F. Varoqueaux (Max-Planck Institute of Experimental Medicine, Göttingen, Germany); anti-carbonic anhydrase 8 (Car8) (guinea pig, 1:500) kindly provided by Dr. M. Watanabe (University School of Medicine, Sapporo, Japan); anti-gephyrin (mouse, 1:1000, #147 011) and anti-VGAT (rabbit, 1:3000, #131 003) purchased from Synaptic Systems (SYSY, Germany); anti-dystrophin (mouse, 1:20, #DYS2-CE-S) and anti-β-dystroglycan (mouse, 1:500, #B-DG-CE) purchased from Leica Biosystem (Buffalo Grove, IL, United States); anti-α-dystroglycan (mouse, 1:100, #05-298, clone VIA4-1, Upstate-Millipore, Germany); anti-S-SCAM/MAGI-2 (rabbit, 1:100, #M2441, Sigma-Aldrich, Germany); anti-bassoon (mouse, 1:3000, #VAM-PS003, clone SAP7F407, Enzo Life Science, East Farmingdale, NY, United States); anti-calbindin (mouse, 1:10000, #300, Swant, Switzerland); anti-GAD65 (mouse, 1:1000, #GAD-6, Developmental Studies Hybridoma Bank, Iowa City, IA, United States). The sections were then rinsed and incubated with the appropriate secondary antibodies, raised either in goat or in donkey, conjugated to one of the following fluorophores: Alexa 488 and Alexa 568 (Molecular Probes, Eugene, OR, United States), or the cyanine-derived Cy3 and Cy5 (Jackson Immunoresearch, West Grove, PA, United States). The sections were rinsed again and coverslipped with Dako fluorescence mounting medium (Dako Italia, Italy).

### Confocal Microscopy and Data Analysis

The sections were analyzed with a laser-scanning confocal microscope (Zeiss LSM5 Pascal, Germany) using the multichannel acquisition mode to avoid fluorescence crosstalk. Quantitative analyses were performed on a minimum of three mice per group. Synaptic structures were analyzed on images acquired with a × 100 oil-immersion objective (1.4 numerical aperture) at a magnification of 8.1 × 10^–3^ μm^2^/pixel, and the pinhole set at 1 Airy unit. The images were processed with the image-analysis program Imaris (release 4.2; Bitplane, Zurich, Switzerland). After segmentation, synapse density was quantified with NIH Fiji:Image J software^1^. In PC-ΔDG mice, quantitative analysis of the number of PCs expressing DG was done with α-DG immunostaining. The number of perisomatic and axo-dendritic synapses was determined by counting manually synaptic clusters at the surface of PCs, some of which were labeled for Car8 or calbindin. Gephyrin clusters were quantified only at axo-dendritic synapses due to the absence of gephyrin at mature perisomatic synapses (Viltono et al., 2008). For *pinceau* analysis, we measured both the area covered by VGAT staining and the mean pixel intensity of the single VGAT-positive *pinceau* using NIH Fiji:Image J software.

### Electron Microscopy

Mice aged 6 months or more were perfused with 1% formaldehyde and 1% glutaraldehyde in PB. The cerebellum was dissected, post-fixed in the same fixative overnight, and the vermis was cut into sagittal sections with a scalpel. The sections were post-fixed in osmium tetroxide (1% in 0.1M cacodylate buffer), dehydrated in ethanol and embedded in Epon-Araldite. Ultrathin sections were collected on copper mesh grids, stained with uranyl acetate and lead citrate and observed with a JEM-1010 and a JEM-1400Flash electron microscope (Jeol, Japan) equipped with a side-mounted sCMOS camera. The number of perisomatic synapses was determined by counting synaptic boutons at the surface of PCs.

#### Immunogold Labeling

Adult mice (aged 3 months) were perfused with 2% formaldehyde and 0.1% glutaraldehyde in sodium acetate buffer, followed by 1 h perfusion with 2% formaldehyde and 0.1% glutaraldehyde in 0.1M borate buffer. Brains were post-fixed in the second fixative solution overnight. Tissue blocks from the cerebellar vermis were freeze-substituted and embedded in Lowicryl HM20. Ultrathin sections were processed for the immunogold method using as secondary antibodies goat Fab fragments coupled to 10 nm colloidal gold particles (Sassoe-Pognetto and Ottersen, 2000).

### Electrophysiology

#### Slice Preparation

Cerebellar slices were routinely prepared from PC-ΔDG and wild-type (c-WT) littermate controls, at different postnatal weeks. Mice were decapitated under halothane anesthesia, and whole brains were rapidly removed and incubated in chilled, oxygenated (95% O_2_, 5% CO_2_) glycerol-based cutting solution (in mM): 2.5 KCl, 2.4 CaCl_2_, 1.2 MgCl_2_, 1.2 NaHPO_4_, 26 NaHCO_3_, 11 glucose, 250 glycerol. Sagittal cerebellar slices (250 μm) were cut at 4°C, using a Vibratome (DSK, Dosaka EM, Kyoto, Japan). Before use, slices were maintained for at least 1 h at room temperature (22–25°C) in oxygenated (95% O_2_, 5% CO_2_) ACSF, containing the following (in mM): 125 NaCl, 2.5 KCl, 1.25 NaH_2_PO_4_, 26 NaHCO_3_, 2 CaCl_2_, 1 MgCl_2_, and 10 glucose, pH 7.35. All recordings were performed at room temperature on slices submerged in ACSF in the recording chamber. The ACSF was perfused at a rate of 1 ml/min.

#### Patch-Clamp Recording

Neurons were visualized at × 640 with Nomarski optics with an upright Zeiss Axioscope microscope. Patch-clamp recordings were obtained using glass electrodes (3–5 MΩ) filled with the following (in mM): 140 Cs-methanesulfonate, 2 MgCl_2_, 10 HEPES, 2 MgATP, 0.5 EGTA; pH 7.3, with CsOH. Neurons were clamped at −70 or 0 mV. Membrane currents, recorded with a patchclamp amplifier (Axopatch 200A; Molecular Devices), were filtered at 2 kHz, digitized (10 kHz), and acquired with Clampex 10 software (Molecular Devices). The stability of the patch was checked by repetitively monitoring the input and series resistance during the experiment, and recordings were discarded when any of these parameters changed by 10%. Data were analyzed offline with Clampfit 10 (Molecular Devices).

### Behavioral Analysis

#### Behavioral Tests Were Performed Blind to the Genotype

##### Inverted screen

PC-ΔDG and littermate controls were placed individually on a cage wire screen about 35 cm above a table. After slowly inverting the screen upside-down to 180°, the ability to maintain a grip was monitored (grip latency) and a maximum score of 120 was given if the animal did not fall. Testing was repeated three times with 10-min inter-trial intervals.

##### Wire suspension

The front paws of the mice were positioned on a horizontal steel wire (0.6 mm thick) suspended at a height of 30 cm above a table. Three trials spaced by a 5-min pause were performed with each trial limited to 60 s duration. The latency to touch the wire with one hind paw were recorded during each trial; a mean score was then calculated. Other qualitive parameters were recorded and a score was attributed corresponding to the best performance achieved within the minute of testing according to the following scale (Helleringer et al., 2018): (0) fell off; (1) clung to the bar with two forepaws; (2) attempted to climb on to the bar besides clinging to it with two forepaws; (3) hung on to the bar with two forepaws and one or both hind paws; (4) hung on to the bar with all four paws with the tail additionally wrapped around the bar; (5) escaped to one of the supports.

##### Rotarod

Motor coordination and learning were evaluated by using a mouse rotarod with adjustable speed and accelerating mode (Ugo Basile, Italy). Mice were habituated to the rod for 2 days prior to the test, by placing them on to non-rotating rod on the first day to test equilibrium (speed: 0 rotation per minute, 0 rmp) and then the second day on the rod rotating at a constant speed of rpm to evaluate basal motor coordination. The fall latency was recorded with a 180 s cut-off duration. Ina second study phase, motor synchronization learning was tested for three consecutive days by placing the mice on the rotating rod with an acceleration protocol (4 to 40 rpm in 5 min). Mice were submitted to five training sessions, one session on the first training day and then two daily sessions during day 2 and day 3. Each session was composed of five successive trials. Between each trial the mouse was placed back in its cage for a minimum of 5 min to recover from physical fatigue. The fall latency recorded during the five trials of a session was averaged for each mouse. Motor learning performance was assessed by comparing the changes in mean fall latency across the five successive sessions in the two genotypes.

### Statistical Analysis

All data are presented as mean ± standard error. Behavioral differences between groups were verified using two-way ANOVAs with repeated measures (training days, trials). Patch-clamp recordings, immunohistochemistry and electron microscopy quantifications were compared using unpaired *t*-test. *p* < 0.05 was used to define statistical significance. Statistics were performed using the Statview 5.0 (SPSS, United States) or GraphPad version 5.0 (Prism) softwares.

## Results

### Dystroglycan Is Essential for Assembly of the Dystrophin–Glycoprotein Complex in Purkinje Cells

**FIGURE 1:**
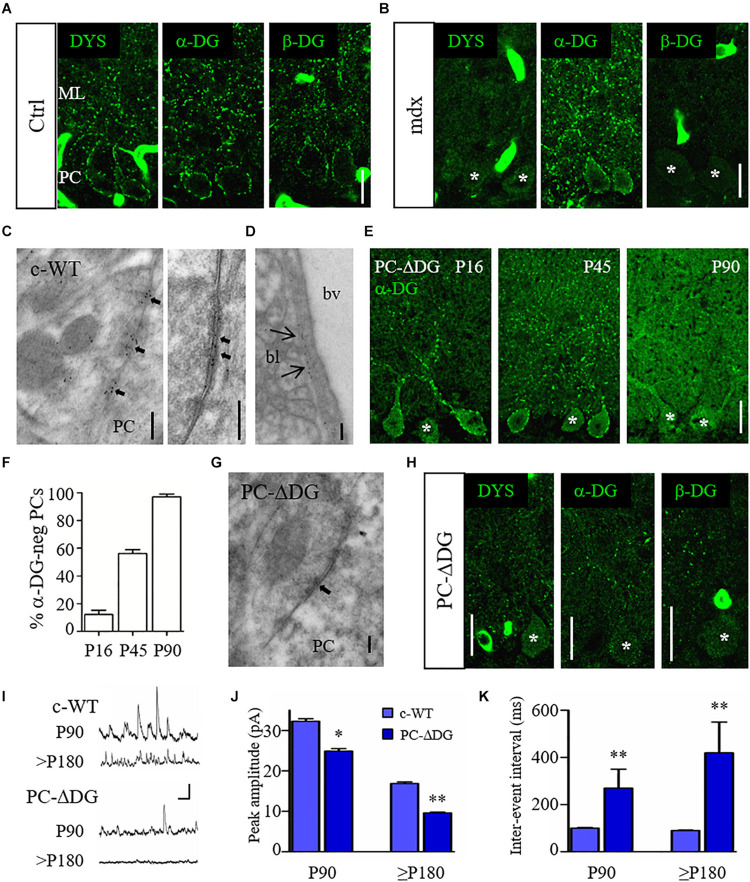
Organization and function of the DGC at GABA synapses. **(A)** Representative confocal images showing immunofluorescence labeling for dystrophin (DYS), α-dystroglycan (DG) and β-DG in adult control (Ctrl) cerebellum. Note punctate staining outlining the cell body of Purkinje cells (PCs) and their dendrites in the molecular layer (ML). **(B)** Immunofluorescence labeling for DYS, α-DG and β-DG in adult *mdx* cerebellum. PCs show labeling for α-DG but are immunonegative for DYS and β-DG. **(C)** Immunogold labeling of a control (c-WT) mouse reveals the presence of α-DG at symmetric synaptic specializations on PCs. The left panel shows an axon terminal contacting the cell body of a PC. Labeling for α-DG is concentrated selectively at the active zones (arrows). The right panel shows a symmetric synapse (arrows) at higher magnification. Note that gold particles mainly localize in the synaptic extracellular space. **(D)** Immunogold labeling for α-DG at the basal lamina (bl) of a blood vessels (bv). **(E)** Immunostaining for α-DG in PC-ΔDG mice of different ages shows the progressive ablation of DG from PCs. Asterisks identify α-DG-negative PCs. **(F)** Quantification of α-DG-negative PCs cells at different ages (*n* = 71 cells at P16, *n* = 176 cells at P45, *n* = 71 cells at P90, 2–4 mice per age). **(G)** Immunogold labeling for α-DG in a PC-ΔDG cerebellum shows an unlabeled symmetric synapse (arrow). **(H)** Representative confocal images of DYS, α-DG and β-DG immunofluorescence in the cerebellum of P90 PC-ΔDG mice. Note the dramatic reduction of cluster density for the three constituents of the DGC. **(I)** Representative traces of sIPSCs recorded from c-WT and PC-ΔDG cells at P90 and P180. Vertical bar: 50 pA; horizontal bar: 250 ms. **(J,K)** Quantitative analysis showing reduced amplitude **(J)** and frequency **(K)** of sIPSCs recorded from PCs of PC-ΔDG mice (*n* = 7–8 cells, two mice) compared to control littermates (c-WT, *n* = 9 cells, two mice). Unpaired *t*-test. ^∗^*p* < 0.05; ^∗∗^*p* < 0.01. Data represent mean ± SEM. Scale bar: 20 μm **(A,B,E,H)**; 200 nm **(C,D,G)**.

To start addressing the role of two major constituents of the DGC, dystrophin and DG, we analyzed specific mouse models in which one of these proteins was missing. We first analyzed *mdx* mice, a murine model of Duchenne muscular dystrophy (DMD) lacking full length (427 kDa) dystrophin. As previously reported (Knuesel et al., 1999; Brunig et al., 2002; Patrizi et al., 2008), labeling of dystrophin, α-DG and β-DG can be detected in large perisomatic and dendritic clusters along PCs (Figure 1A), where these molecules co-localize precisely with GABA_A_Rs and NL2 (Patrizi et al., 2008). Surprisingly, the lack of dystrophin in *mdx* mice affected the synaptic localization of β-DG without altering the localization of α-DG (Figures 1A,B). Thus, α-DG and β-DG have different dependencies on dystrophin in GABAergic synapses.

We then evaluated the role of DG at the GABAergic post-synaptic compartment. First, we analyzed the distribution of DG using immunogold labeling and electron microscopy with an antibody against α-DG. Ultrastructural analyses confirmed that in c-WT cerebella labeling for α-DG was present in symmetric synaptic specializations of PCs (Figure 1C, left). Gold particles were mainly localized in the synaptic cleft, consistent with an extracellular localization of α-DG (Figure 1C, right). Moreover, labeling for α-DG was observed in the basal lamina surrounding brain capillaries (Figure 1D) (Nickolls and Bonnemann, 2018). Then, we generated conditional DG KO mice by crossing mice harboring loxP sites in *Dag1* gene (*Dag1* ^*loxP/loxP*^) (Cohn et al., 2002) with the L7Cre transgenic line (L7Cre ^*Tg/Tg*^), which exhibits a selective Cre recombinase expression in PCs (PC-ΔDG; L7Cre ^*Tg/+*^, *Dag1* ^*loxP/loxP*^). PC-ΔDG mice appeared healthy and showed no obvious neurological abnormalities (not shown). Interestingly, we found that α-DG immunoreactivity was gradually lost starting in the second and third postnatal weeks. Thus, in P16 mice labeling of PCs had a mosaic-like pattern, characterized by immunopositive (α-DG-pos) and immunonegative (α-DG-neg) cells, which in several cases were adjacent (Figure 1E). At these early stages, only a small percentage of PCs had lost DG immunoreactivity, whereas at P45 more than 50% of PCs were α-DG-neg (Figure 1F). By the age of P90, the large majority of PCs were DG-neg (Figure 1F). This mosaic-like pattern is consistent with the asynchronous expression of L7 in different PCs (Barski et al., 2000; Briatore et al., 2010). Immunogold labeling in P90 PC-ΔDG mice showed an almost complete elimination of α-DG from synaptic profiles, where gold particles were found only occasionally (Figure 1G), confirming the selective ablation of DG from PCs. Notably, ablation of DG was enough for the complete disappearing of dystrophin from post-synaptic compartments (Figure 1H), demonstrating that the synaptic localization of dystrophin depends on DG *in vivo*, as previously reported in forebrain neurons (Levi et al., 2002; Früh et al., 2016).

We then evaluated the functional consequences of DG loss from PCs in P90 mice, when the majority of PCs were α-DG-neg. Spontaneous inhibitory post-synaptic currents (IPSCs) measured from PCs by patch-clamp recordings in acute cerebellar slices revealed a significant reduction of both the amplitude and frequency of iPSCs in PC-ΔDG mice compared to c-WT littermate controls (Figures 1I–k). This difference became even stronger in older PC-ΔDG mice (Figures 1I–k), suggesting that deletion of DG causes a progressive decrease in the number of functional synapses in the cerebellum.

These data indicate that the DGC is important for inhibitory synapse organization and function. Moreover, mutation of selective DGC components differentially affects the molecular organization of GABAergic synapses.

### Dystroglycan Promotes the Clustering of GABAergic Post-synaptic Components

**FIGURE 2:**
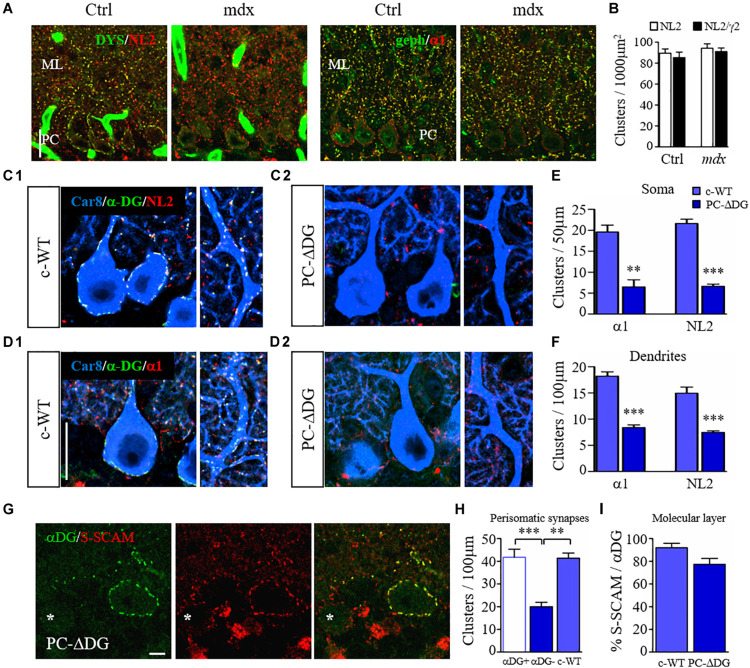
Dystroglycan is required for clustering of post-synaptic proteins. **(A)** Organization of GABAergic post-synaptic molecules in *mdx* and control (Ctrl) littermates. **(A_1_)** Double labeling for dystrophin (DYS) and neuroligin 2 (NL2). Note that clustering of NL2 is not affected by the lack of DYS in *mdx* mice. **(A_2_)** double labeling for gephyrin (geph) and GABA_A_Rα1 reveals no obvious difference between control and mutant mice. Note that gephyrin clusters are normally not present at perisomatic synapses of PCs. ML: molecular layer. **(B)** Quantification showing similar densities of NL2 and GABA_A_Rγ2 clusters in the molecular layer of Ctrl and *mdx* mice (*n* = 3–4 mice per genotype). **(C)** Triple immunofluorescence labeling for Car8 (a marker of PCs), α-DG and NL2 in c-WT **(C_1_)** and PC-ΔDG mice aged ∼3 months **(C_2_)**. In c-WT mice, NL2 colocalizes with α-DG (triple labeling results in white puncta). In contrast, NL2 clusters are almost completely absent from PCs of PC-ΔDG mice. The NL2-positive clusters visible outside of PCs (red puncta) represent synapses on cerebellar interneurons, which do not normally express DG. **(D)** Triple labeling for Car8, α-DG and GABA_A_Rα1 in c-WT and PC-ΔDG mice. GABA_A_Rα1 colocalizes with α-DG in c-WT PCs **(D_1_)**, whereas GABA_A_Rα1 clusters are almost completely absent from PCs of PC-ΔDG mice **(D_2_)**. Quantitative analysis showing the density of somatic **(E)** and axodendritic **(F)** GABA_A_Rα1 and NL2 clusters in control and PC-ΔDG mice (*n* = 3 mice per genotype). **(G)** Representative confocal images showing immunofluorescence labeling for α-DG and S-SCAM in a P90 PC-ΔDG cerebellum. S-SCAM colocalizes precisely with α-DG in a α-DG-positive PC, whereas the density of S-SCAM clusters is reduced in α-DG-negative PC (asterisk). **(H)** Quantitative analysis of the density of perisomatic S-SCAM clusters does not show any difference between c-WT and α-DG-positive PCs (αDG +). In contrast, S-SCAM is significantly downregulated in α-DG-negative PCs (αDG–) compared to c-WT littermates (*n* = 8–10 cells per genotype, four mice per genotype). **(I)** The percentage of S-SCAM clusters colocalized with α-DG in the molecular layer was similar in PC-ΔDG and c-WT mice. Unpaired *t*-test. ^∗∗^*p* < 0.01; ^∗∗∗^*p* < 0.001. Data represent mean ± SEM. Scale bar: 20 μm **(A,C,D)**, 5 μm **(G)**.

To understand how the DGC organizes GABAergic synapses, we used immunofluorescence with antibodies raised against post-synaptic proteins. Inhibitory synapses onto PCs express a homogenous repertoire of post-synaptic molecules, including GABA_A_Rs with the α1 and γ2 subunits, NL2 and gephyrin, together with dystrophin and DG (Patrizi et al., 2008). We therefore analyzed the clustering organization of NL2, GABA_A_Rs and gephyrin in *mdx* and control mice. Surprisingly, these analyses failed to reveal any significant difference between the two genotypes (Figures 2A,B), suggesting that dystrophin is not an absolute requirement for clustering of GABAergic post-synaptic molecules in PCs.

We then analyzed the molecular organization of GABAergic synapses in PCs of PC-ΔDG mice. In control condition, α-DG precisely co-localized with GABA_A_Rα1 and NL2 (Patrizi et al., 2008) (Figures 2C_1_,D_1_). In contrast, clustering of both NL2 and GABA_A_Rα1 was severely altered in α-DG-neg PCs (Figures 2C_2_,D_2_). Quantitative analyses showed an extensive ablation of NL2 and GABA_A_Rα1 in the somatic and dendritic compartments of DG-mutant PCs compared to control littermates (Figures 2E,F). The sporadic puncta that remained detectable along the PC profiles of PC-ΔDG mice (Figures 2C_2_,D_2_) generally had a small size and a weak fluorescence intensity, suggesting impaired aggregation of post-synaptic proteins. Accordingly, the density of dendritic gephyrin clusters was also significantly reduced in PC-ΔDG mice (c-WT: 16.1 ± 1.6 clusters/100 μm; PC-ΔDG: 8.6 ± 0.8; *n* = 3 mice; *p* = 0.0059). These results indicate that selective ablation of DG from individual PCs strongly affects GABAergic post-synaptic constituents.

**FIGURE 3:**
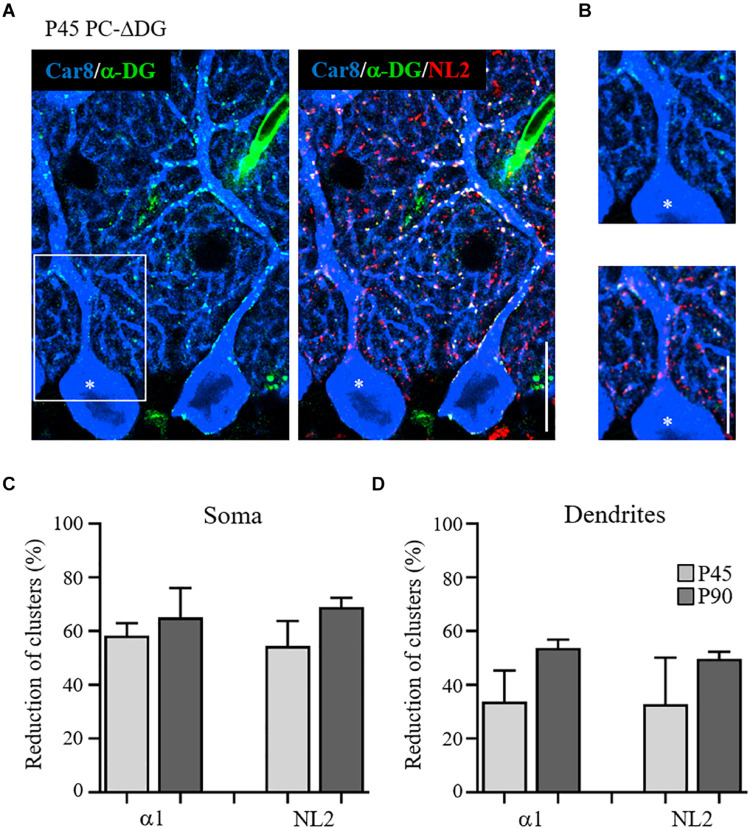
Gradual disappearance of α-dystroglycan from Purkinje cells. **(A)** Triple immunofluorescence labeling for Car8, α-DG and NL2 in a P45 PC-ΔDG cerebellum showing adjacent α-DG-positive and α-DG-negative (asterisk) PCs. In the α-DG-positive PC, α-DG and NL2 colocalize precisely at the perisomatic and dendritic clusters, whereas in the α-DG-negative PC there is a somatodendritic gradient in the loss of α-DG and NL2-positive puncta. **(B)** White box insert shows the residual presence of α-DG and NL2 clusters along the dendritic domain of the α-DG-negative PC. Quantitative analysis showing the reduction of the GABA_A_Rα1 and NL2 perisomatic **(C)** and dendritic **(D)** clusters at P45 and P90 (*n* = 3 mice per genotype). Data represent mean ± SEM. Scale bar: 15 μm.

Similar results were obtained by comparing α-DG-pos and α-DG-neg PCs in younger (P45) animals (Figure 3). Interestingly, there was a gradient in the elimination of DG from the cell body and the dendrites of PCs, that was mirrored by a gradual loss of NL2 (Figure 3A). At these early stages, rare NL2 clusters lacking α-DG could be identified along PC dendrites (Figure 3B), likely representing a transient phase of post-synaptic rearrangement. Quantification at P45 and P90 revealed that conditional knockout of DG caused a disappearance of almost 60% of perisomatic NL2 and GABA_A_Rα1 clusters at both ages (Figure 3C). On the other hand, the reorganization of axodendritic synapses was slower, resulting in a ∼50% cluster reduction only at P90 (Figure 3D). These results indicate that perisomatic synapses are more susceptible to ablation of DG. It is tempting to link this to the fact that mature perisomatic synapses lack gephyrin (Viltono et al., 2008).

S-SCAM is a scaffolding protein essential for the clustering of synaptic receptors and the dynamic turnover of synaptic components (Danielson et al., 2012). At GABAergic synapses, S-SCAM interacts with key post-synaptic molecules, such as β-DG and NL2 (Sumita et al., 2007). We therefore analyzed how the absence of DG affects S-SCAM localization in PCs. In control mice, we noticed that the majority of S-SCAM-positive puncta co-localized with α-DG, suggesting a preferential association of S-SCAM with GABAergic synapses. Interestingly, in PC-ΔDG mice α-DG-neg PCs showed a significant downregulation of S-SCAM clusters, whereas α-DG-pos PCs did not differ from littermate controls (Figures 2G,H). In the molecular layer, practically all S-SCAM clusters were associated with DG clusters, suggesting that their density was strongly reduced after ablation of DG (Figure 2I). These data indicate that in PCs S-SCAM localization at GABA synapses requires DG.

All together, our findings indicate that DG is essential for organizing GABAergic post-synaptic assemblies. Deletion of DG dramatically affects all major GABAergic post-synaptic components, including GABA_A_Rs, the cell adhesion molecule NL2 and the scaffolding proteins S-SCAM and gephyrin.

### Dystroglycan Is Required for GABAergic Innervation

**FIGURE 4:**
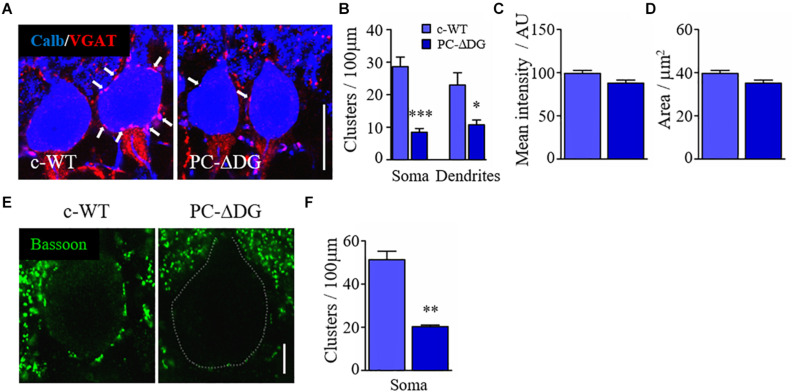
Dystroglycan is required for GABAergic innervation. **(A)** Double immunofluorescence labeling for VGAT and calbindin (calb) showing the perisomatic arrangement of GABAergic presynaptic terminals (white arrows) in PCs of control (c-WT) and PC-ΔDG mice aged ∼3 months. **(B)** Quantitative analysis showing that perisomatic appositions by VGAT-positive elements are dramatically reduced in PCs of PC-ΔDG mice. **(C,D)** Quantitative analysis showing no differences in the *pinceau* mean intensity and mean area between c-WT and PC-ΔDG mice (*n* = 3–5 mice per genotype). **(E)** Immunofluorescence labeling showing the arrangement of bassoon-positive clusters surrounding PCs in c-WT and PC-ΔDG mice aged ∼3 months. **(F)** Quantitative analysis showing the significant reduction of bassoon-positive perisomatic clusters in PCs of PC-ΔDG mice (*n* = 3–4 mice per genotype). Unpaired *t*-test, *p < 0.05; **p < 0.01; ***p < 0.001.

Both DG and NL2 can bind to presynaptic NRXs (Sugita et al., 2001), suggesting that these molecules may play a role in *trans*-synaptic adhesion. Therefore, we decided to investigate to what extent the deletion of DG, and the resulting loss of NL2, affects presynaptic GABAergic innervation. To evaluate the presynaptic compartment, we used an antibody raised against the vesicular inhibitory amino acid transporter (VGAT), which is responsible for GABA uptake and storage in synaptic vesicles. We observed a significant reduction in the density of VGAT-positive terminals contacting PCs (Figure 4A), both in the somatic and dendritic domains (Figure 4B), suggesting that the absence of DG affects GABAergic afferents. Notably, the organization of the *pinceau* at the axon initial segment of PCs was not overtly altered in PC-ΔDG mice (Figure 4A); in fact both the mean labeling intensity and the overall area of the VGAT-positive terminals at the *pinceau* were indistinguishable in the two genotypes (Figures 4C,D), consistent with the fact that the *pinceau* lacks the protein machinery typical of GABAergic synapses (Iwakura et al., 2012).

**FIGURE 5:**
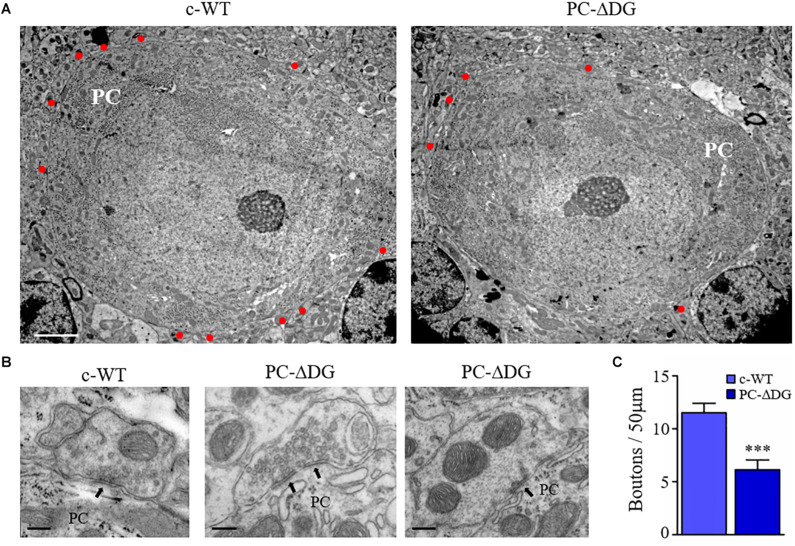
GABAergic synapse elimination in PC-ΔDG mice. **(A)** Representative electron micrographs showing PCs of c-WT and PC-ΔDG mice. Red dots identify GABAergic axon terminals establishing symmetric synaptic contacts. **(B)** Perisomatic synapses shown at higher magnification. Arrows point to symmetric synaptic specializations. **(C)** Quantitative analysis showing a significant reduction of axon terminals contacting PC somas in PC-ΔDG mice compared with c-WT (*n* = 8–10 PCs per genotype). Unpaired *t*-test. ^∗^*p* < 0.05; ^∗∗^*p* < 0.01; ^∗∗∗^*p* < 0.001. Scale bar: 2 μPC-Dm **(A)**, 200 nm **(C)**.

The observations reported above may reflect diminished expression of VGAT in presynaptic terminals and/or reduced GABAergic innervation of PCs. To distinguish between these possibilities, we investigated the expression of another presynaptic protein, bassoon, and we found a dramatic decrease in the density of perisomatic bassoon-positive puncta in PC-ΔDG PCs (Figures 4E,F). Because bassoon is also present in excitatory synapses, we did not assess its expression in the molecular layer due to the very high density of puncta. Finally, we used electron microscopy to analyze the density of presynaptic boutons establishing contacts with the cell body of PCs. This ultrastructural analysis revealed a remarkable (almost 50%) decrease of perisomatic contacts in PCs of PC-ΔDG mice (Figures 5A–C). The axon terminals establishing these symmetric junctions were very similar to those seen in control animals, although some were characterized by a paucity of presynaptic vesicles (Figure 5B). Heterologous contacts made by climbing fibers or other glutamatergic axons were not observed. Thus, it is likely that the residual symmetric synapses on mutant PCs are made by molecular layer interneurons. Together, these data indicate that DG plays a pivotal role in *trans*-synaptic signaling required for the maintenance of GABAergic synapses in PCs.

**FIGURE 6:**
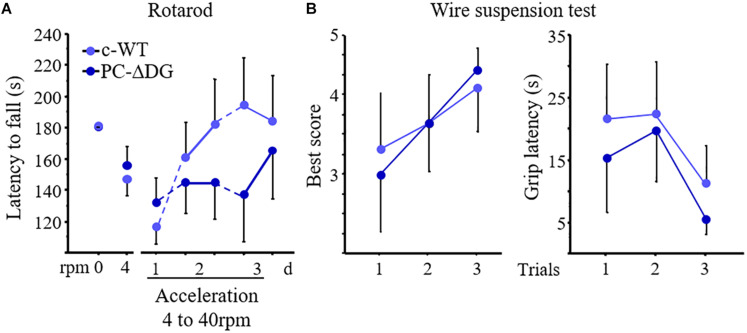
Motor learning defects in PC-ΔDG mice. **(A)** The latency to fall from rotarod does not differ between PC-ΔDG and c-WT littermates during the accommodation trials at 0 and 4 rotations per minute (rpm). In contrast, motor-coordination learning significantly decreases in PC-ΔDG compared to c-WT mice during the acceleration trials (4 to 40 rpm) developed over 3 days (d) (Two-way ANOVA, Genotype x session: *p* < 0.05, *n* = 9 mice per group). **(B)** No differences in the best score (left panel) and in the latency to grip the wire (right panel) in the wire suspension test between PC-ΔDG and c-WT controls (*n* = 9 mice per group). Data represent mean ± SEM.

Finally, we investigated whether DG mutants have impaired motor performances and motor learning. PC-ΔDG mice showed no obvious signs of tremor or ataxia (not shown). In contrast, the accelerating rotarod test revealed significantly impaired motor learning performance in the mutants (Figure 6A). Indeed, during the accommodation period (rpm 0 and rpm 4) the two groups showed a comparable fall latency, suggesting unaltered static equilibrium and basal dynamic coordination, respectively. In contrast, during the acceleration phase (day 1 to day 3) the motor abilities improved in control but not in mutant mice (Figure 6A). We did not find significant differences between PC-ΔDG and c-WT mice in the inverted screen (not shown) and wire suspension tests (Figure 6B), thus, confirming that muscle strength and basal motor coordination were not affected in PC-ΔDG. These data suggest that selective deletion of DG from PCs causes a severe alteration of GABAergic synaptic compartments, leading to impaired motor synchronization learning.

## Discussion

**FIGURE 7:**
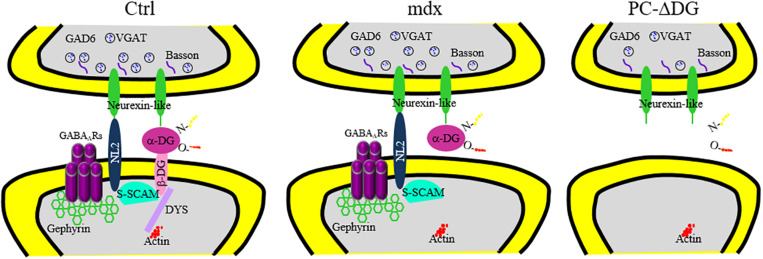
Reorganization of major pre-and post-synaptic proteins after ablation of dystroglycan from PCs. Summary of the distribution of essential GABAergic synaptic components in PCs of control (Ctrl, left panel), *mdx* (middle panel) and PC-ΔDG mice (right panel). Briefly, α- and β-dystroglycan (DG) bridge presynaptic neurexin (NRX) or NRX-like proteins with post-synaptic structural proteins, such as dystrophin (DYS) and S-SCAM. In turn, S-SCAM establishes a link between neuroligin 2 (NL2) and β-DG. Note that both NL2 and α-DG are capable of interacting with presynaptic NRX or NRX-like proteins. GABA_A_ receptors are stabilized by a submembranous lattice of gephyrin by direct interaction. In *mdx* mice, the absence of dystrophin alters the synaptic localization of β-DG. However, α-DG and other post-synaptic proteins are largely unaffected. Ablation of DG in PC-ΔDG mice causes impaired clustering of post-synaptic proteins and GABAergic synaptic instability.

DG is a central component of the DGC, which links the cytoskeleton to the extracellular matrix in different cell types. Mutations affecting DGC components lead to muscular dystrophies with variable degrees of central nervous system involvement (Barresi and Campbell, 2006; Godfrey et al., 2011). Deletion of DG selectively from neurons causes subtle defects, such as altered long-term potentiation in the hippocampus (Satz et al., 2010), but the precise mechanisms by which DG regulates synaptic function and plasticity remain unknown. In this study, we dissected DG function in cerebellar PCs. The major findings are summarized in Figure 7. Briefly, we show that DG is a crucial organizer of GABA synapses, linking structural scaffolding proteins with synaptic cell-adhesion molecules. In particular, selective ablation of DG from PCs disrupts the clustering of major constituents of the GABAergic post-synaptic protein network, causing reduced GABAergic currents and delayed learning of motor synchronization. The drastic alteration of the post-synaptic compartment in DG-deprived PCs is accompanied by a severe reduction of GABAergic innervation, suggesting that DG is required for synapse maintenance. Remarkably, these synaptic alterations were not present in *mdx* mice that retain α-DG at synapses, supporting the idea that the stability of GABAergic synapses in PCs depends on extracellular interactions mediated by α-DG. Thus, α-DG is a novel secreted synaptic organizer that localizes in the synaptic cleft and mediates *trans*-synaptic interactions in a subset of GABAergic synapses.

### Dystroglycan Is Required for Clustering of Post-synaptic Proteins

A key finding of our study is that deletion of DG from PCs impaired the post-synaptic accumulation of major post-synaptic proteins, such as NL2, S-SCAM, gephyrin and GABA_A_Rs. Historically, it has been assumed that a subset of inhibitory synapses is dependent upon the presence of NL2 or the gephyrin- and NL2-binding guanine nucleotide exchange factor (GEF) collybistin (Patrizi et al., 2008; Krueger et al., 2012). However, to our knowledge, no study has addressed the role of NL2 or other cell-adhesion molecules in the clustering of the DGC complex at central synapses. Our data suggest that in PCs DG may act upstream of NL2 as a nucleation site that governs the assembly of the GABAergic post-synaptic specialization. This is a particularly dramatic effect in light of the fact that neither synaptic GABA_A_Rs nor collybistin are required for post-synaptic clustering of NL2 (Patrizi et al., 2008; Poulopoulos et al., 2009; Frola et al., 2013).

The clustering of NL2 by DG may appear at first surprising, because NL2 also binds presynaptically to NRX, and it is generally assumed that NRX-NL interactions represent a key step in the developmental assembly of synapses (Sudhof, 2008; Shen and Scheiffele, 2010; Siddiqui and Craig, 2011; Krueger et al., 2012). However, we have previously demonstrated that NRX expression is developmentally regulated at GABA synapses in PCs. Specifically, NRX is associated with GABAergic synapses during early postnatal development, and is downregulated in mature circuits (Pregno et al., 2013). This raises the intriguing possibility that NRX-NL2 interactions could be involved in early stages of synaptic adhesion (Craig and Kang, 2007), whereas DG stabilizes newly formed synapses. Unfortunately, this hypothesis cannot be verified in our mouse model. In fact, due to the late temporal profile of L7Cre recombinase expression in PCs (Barski et al., 2000; Briatore et al., 2010), the deletion of DG starts around the time when NRX is downregulated at GABAergic synapses (Pregno et al., 2013).

Our findings suggest that distinct components of the DGC play exquisitely specific roles in regulating the GABAergic post-synaptic protein network. In particular, the analysis of *mdx* mice reveals that dystrophin is not essential for post-synaptic clustering of NL2, GABA_A_Rs and gephyrin. Similarly, in hippocampal CA1 the absence of dystrophin did not abolish synaptic clustering of NL2 *per se*, although the authors reported a complex pattern of alterations in the distribution of pre- and post-synaptic proteins of inhibitory synapses in dystrophin-deficient mice, most likely reflecting a rearrangement of the GABAergic synaptic network (Krasowska et al., 2014). Our results contrast with previous analyses revealing selective deficits in the synaptic clustering of GABA_A_Rs, but not gephyrin, in the cerebellum and amygdala of *mdx* mice (Knuesel et al., 1999; Sekiguchi et al., 2009). However, the selective loss of GABA_A_R, but not gephyrin, clusters is surprising, also considering that deletion of GABA_A_Rs from PCs causes a severe defect in the clustering of gephyrin (Kralic et al., 2006; Patrizi et al., 2008), without affecting dystrophin and DG (Patrizi et al., 2008). It is important to notice that dystrophin is required for normal GABAergic function in PCs, CA1 pyramidal cells and amygdala neurons (Anderson et al., 2003; Vaillend et al., 2004; Kueh et al., 2008; Sekiguchi et al., 2009). One possible explanation, which could reconcile our data with those of Knuesel et al. (1999), is that dystrophin contributes to stabilize post-synaptic GABA_A_Rs by regulating the trafficking of peri/extra-synaptic receptors (Vaillend and Chaussenot, 2017) and that loss of dystrophin causes subtle effects not readily detected by our sensitive immunofluorescence procedure.

While post-synaptic changes in *mdx* mice were minor, the selective deletion of DG from PCs caused a dramatic decrease of both GABA_A_Rs and gephyrin clusters, accompanied by a significant downregulation of sIPSC frequency and amplitude. Our results differ from those of a recent study in which conditional deletion of DG from hippocampal pyramidal neurons, under the Nex promoter, only lead to minor alterations in GABAergic protein clustering (Früh et al., 2016). One possible explanation for this discrepancy is that the arrangement of the DGC may differ in PCs and telencephalic pyramidal neurons. For example, the DGC mainly localizes in perisomatic synapses of pyramidal neurons (Knuesel et al., 1999), whereas in PCs all perisomatic and axo-dendritic GABAergic synapses contain dystrophin and DG (Patrizi et al., 2008; Briatore et al., 2010). Früh et al. (2016) also reported a selective loss of GABAergic synapses established by cholecystokinin (CCK)-positive basket cells after deletion of DG, but failed to detect differences in the density of VGAT-positive boutons, suggesting that overall GABAergic innervation was normal in their mutant. This raises the possibility that compensation by other basket cell terminals may have masked post-synaptic effects caused by DG deletion. However, it remains possible that DG plays a somewhat different role in different types of GABAergic synapses, requiring a better understanding of the neuron type-specific function of DG in *trans*-synaptic signaling.

### Dystroglycan Is Required for GABAergic Synapse Maintenance

In addition to the disruption of the post-synaptic compartment, ablation of DG from PCs leads to a *bona fide* elimination of synapses. In particular, deletion of DG resulted in reduced GABAergic innervation of PCs, as evidenced by both immunofluorescence and electron microscopy, accompanied by a prominent downregulation of sIPSC frequencies. This is a particularly dramatic effect, considering that genetic ablation of synaptic adhesion proteins frequently does not result in synapse loss (Piechotta et al., 2006). For example, single, double or triple conditional knockout of NL1, NL2, and NL3 from PCs selectively decreases the amplitude of IPSCs in PCs but does not affect inhibitory synapse density (Zhang et al., 2015).

Our findings are reminiscent of the situation described for cerebellin 1 precursor protein (Cbln1), a glycoprotein of the complement C1q-related family secreted from granule cells axons. Cbln1 mediates the formation and maintenance of glutamatergic synapses between parallel fibers and PC spines by binding to presynaptic NRX and to post-synaptic GluD2 (Uemura et al., 2010; Joo et al., 2011). Although the presynaptic binding partner of DG remains to be identified, our findings suggest that DG, similar to Cbln1, mediates *trans*-synaptic interactions that are essential for synapse maintenance. In particular, the extracellularly located α-DG could establish a link between the post-synaptic site, through its binding to transmembrane β-DG, and the presynaptic compartment, most likely through a NRX-like molecule that remains to be identified (Figure 7). Thus, α-DG joins the list of secreted synaptic organizers that reside in the extracellular matrix and act bidirectionally to coordinate selective interactions between the pre- and post-synaptic compartments (Johnson-Venkatesh and Umemori, 2010; Sassoe-Pognetto and Patrizi, 2017; Yuzaki, 2018). In the future, it will be important to implement transcriptional and proteomic analyses, such as single-cell RNA sequencing (Carter et al., 2018; Peng et al., 2019) and immunoprecipitation of α-DG from murine cerebellum in combination with mass spectrometry to identify new bridge molecules.

### How Does the DGC Organize GABAergic Synapses?

Comparison of the synaptic phenotype of and *mdx* mice (Figure 7) offers the opportunity for a molecular dissection of the role of individual DGC constituents. One possible mechanism by which the DGC may contribute to stabilize GABAergic post-synaptic proteins and maintain GABA synapses involves intracellular interactions mediated by the multi-PDZ scaffold S-SCAM, which links β-DG to the NL2 cytoplasmic tail (Sumita et al., 2007) (Figure 7). S-SCAM, which has been implicated in the assembly of GABAergic synapses (Woo et al., 2013), also appears to form bridges between dystrophin and SynArfGEF, a guanine exchange factor for the Arf6 small GTPases, thereby establishing potential links between distinct subdomains of the inhibitory post-synaptic specialization (Fukaya et al., 2011). However, synaptic clustering of NL2, gephyrin and GABA_A_Rs was not overtly modified in *mdx* mice, which lack dystrophin and β-DG (Figure 7), suggesting that intracellular interactions mediated by β-DG are not required for assembly of the GABAergic post-synaptic protein network and for synapse maintenance. Rather, our results indicate that persistence of α-DG in *mdx* mice is sufficient for preserving a largely intact post-synaptic specialization. We propose that the heavily glycosylated α-DG subunit may stabilize GABAergic synapses via extracellular interactions with cell-surface proteins containing laminin G-like domains (Barresi and Campbell, 2006). The extensive glycosylation of α-DG and the broad range of extracellular ligands suggest that this protein has the potential to act as a multidomain connector that brings together distinct complexes of proteins that act synergistically within the synaptic specialization.

NRX is a likely synaptic α-DG binding partner, as this protein can establish interactions with both α-DG and NL2 (Sugita et al., 2001). However, NRX is downregulated in mature cerebellar GABAergic synapses (Pregno et al., 2013), suggesting that α-DG could interact with other presynaptic molecules, potentially including NRX-like cell-surface proteins (Missler and Sudhof, 1998). One example is contactin associated protein-like 4 (CNTNAP4), also known as CASPR4, that has been localized presynaptically in developing murine cortical interneurons (Karayannis et al., 2014). Future studies will be needed to confirm the absence of NRX in mature GABAergic synapses onto PCs as well as to identify novel DG presynaptic binding partners.

### Dystroglycan Is a New Organizer of Inhibitory Synapses

The present findings reveal that DG is a new organizer of a subset of GABAergic synapses, and support the idea that secreted proteins can act bidirectionally to coordinate interactions between the pre- and post-synaptic compartments (Yuzaki, 2018). The selective localization of DG and other DGC constituents in selected types of GABAergic synapses also suggests that these molecules are part of a molecular signature that contributes to generate synapse specificity (Sassoe-Pognetto and Patrizi, 2017). Indeed, GABAergic synaptic specializations differ molecularly and functionally in different interneuron subtypes throughout the brain (Contreras et al., 2019), but our understanding of the distribution and function of selective *trans*-synaptic adhesion systems at inhibitory synapses is still incomplete. A recent study demonstrated that distinct secreted synaptic organizers selectively drive cortical GABAergic synapse formation in distinct compartments of their post-synaptic cells. For example, Cbln4 was shown to be essential for dendritic targeting of GABAergic axons, whereas leucine-rich repeat LGI family member 2 (LGI2) emerged as a promising candidate to regulate the development of perisomatic inhibitory synapses (Favuzzi et al., 2019). Together, these data support the emerging notion of a cell type-specific molecular code of GABAergic synapses, which results from the presence of multiple *trans*-synaptic adhesion systems characterized by partially overlapping distributions and variable degrees of redundancy.

## Data Availability Statement

All datasets generated for this study are included in the article/supplementary material.

## Ethics Statement

The animal study was reviewed and approved by Italian Ministry of Health (Department of Public Veterinary Health) and the ethical committee of Turin University.

## Author Contributions

FB, GP, AP, CV, and MS-P conceived and designed the project. FB, GP, and EF performed morphological analyses. FB, GP, EF, and AP analyzed morphological data. SD and MD performed and analyzed electrophysiology analysis. CV performed and analyzed behavioral data. AP and MS-P wrote the manuscript. All authors edited the manuscript.

## Conflict of Interest

The authors declare that the research was conducted in the absence of any commercial or financial relationships that could be construed as a potential conflict of interest.

## Footnotes

1 https://imagej.net/Fiji
